# Comparison of Glutathione, Retinol and α- and γ-Tocopherols Concentrations Between Children with and Without Epilepsy: A Single-Center Case–Control Study

**DOI:** 10.3390/brainsci15060655

**Published:** 2025-06-18

**Authors:** Izabela Szołtysek-Bołdys, Wioleta Zielińska-Danch, Łucja Gajowska, Ilona Kopyta, Beata Sarecka-Hujar

**Affiliations:** 1 Department of General and Inorganic Chemistry, Faculty of Pharmaceutical Sciences in Sosnowiec, Medical University of Silesia in Katowice, 41-200 Sosnowiec, Poland; iboldys@sum.edu.pl (I.S.-B.); wzdanch@sum.edu.pl (W.Z.-D.); 2 NZOZ Central Laboratory in Bytom, 41-902 Bytom, Poland; lucjaml@interia.pl; 3 Department of Child Neurology, Faculty of Medical Sciences in Katowice, Medical University of Silesia in Katowice, 40-752 Katowice, Poland; ilonakopyta@autograf.pl; 4 Department of Basic Biomedical Science, Faculty of Pharmaceutical Sciences in Sosnowiec, Medical University of Silesia in Katowice, 41-200 Sosnowiec, Poland

**Keywords:** anti-seizure medications, epilepsy, glutathione, tocopherol, retinol, oxidative stress

## Abstract

Background: Oxidative stress is associated with the pathogenesis of epilepsy. Long-term treatment with anti-seizure medications (ASMs) may reduce antioxidant levels, which consequently impairs the brain’s ability to counteract oxidative damage. This study aimed to assess the concentrations of selected antioxidants (i.e., glutathione, retinol, and α- and γ-tocopherols) in children with epilepsy treated with polytherapy. Methods: The study included 21 children with epilepsy treated with ≥2 ASMs for at least 6 months (mean age 7.1 ± 4.4 years) and 23 control children without epilepsy (mean age 7.4 ± 3.9 years). Both groups were recruited at the Department of Pediatric Neurology, the Medical University of Silesia in Katowice (Poland). The concentrations of glutathione, retinol, and α- and γ-tocopherols were determined in blood serum by HPLC. The antioxidant levels were compared between sex and age subgroups of individuals with epilepsy. Results: In the group of individuals with epilepsy, the percentage of females was 38% and in the control group it was 30%. There were no differences in antioxidant levels between female and male individuals with epilepsy, nor between younger epileptic children (0–6 years) and older children (>6 years). Individuals with epilepsy had significantly lower glutathione levels than the control group (1.5 ± 0.3 µmol/L vs. 2.4 ± 1.2 µmol/L, respectively, *p* < 0.001). In turn, the ratios of both α-tocopherol/glutathione and γ-tocopherol/glutathione were higher in individuals with epilepsy than in the control group (*p* = 0.042 and *p* = 0.004, respectively). Individuals with epilepsy taking ASM combinations other than valproic acid (VPA) and levetiracetam (LEV) had a lower level of both retinol and glutathione than individuals on VPA and LEV treatment (for retinol 0.44 ± 0.13 µmol/L vs. 0.6 ± 0.1 µmol/L, respectively, *p* = 0.047, and for glutathione 1.3 ± 0.3 µmol/L vs. 1.8 ± 0.3 µmol/L, respectively, *p* = 0.003). In the individuals with epilepsy, the level of α-tocopherol decreased with age (r = −0.505, *p* = 0.019). In turn, in the control group, the levels of retinol and γ-tocopherol increased with age (r = 0.573, *p* = 0.004 and r = 0.461, *p* = 0.027, respectively). Conclusions: Glutathione levels significantly differed between children with and without epilepsy. The concentration of α-tocopherol decreased with age in pediatric individuals with epilepsy. The levels of both retinol and glutathione were higher in individuals with epilepsy taking VPA and LEV treatment compared to individuals on ASMs combination other than VPA and LEV.

## 1. Introduction

Epilepsy is one of the most common neurological disorders worldwide, affecting approximately 50 million people [1,2]. The highest number of new cases occurs in older people and children [3,4,5]. It is characterized by recurrent, often spontaneous seizures [6]. Epileptic patients are at a risk of developing comorbidities [7]. The cause of seizures is high-frequency discharges that arise as a result of the excitation of nerve cells in the brain [8]. The disease significantly affects the lifestyle of the patients, causing disturbances in daily functioning, such as physical limitations and changes in quality of life. There are often problems with acceptance in peer groups and associated loneliness [9]. The course of epilepsy depends on the frequency of attacks, psychosocial aspects, and the success of treatment. Epilepsy treatment is a long-term process that may negatively affect the patient’s health through the risk of adverse effects of medications taken. The drugs used for anti-seizure treatment can be categorized into two main groups based on their interaction with cytochrome P450 (CYP) isoenzymes. The first category comprises drugs that either induce or inhibit these enzymes, such as carbamazepine (CBZ), phenobarbital (PB), phenytoin (PHT), and primidone (PRM), which are known to induce CYP isoenzymes. Conversely, valproic acid (VPA) is recognized for its inhibitory effects on these enzymes. The last category includes anti-seizure medications (ASMs) that do not influence CYP activity, including gabapentin (GBP), levetiracetam (LEV), vigabatrin (VGB), and topiramate (TPM). In general, monotherapy is preferred as the initial treatment for childhood epilepsy due to comparable efficacy and better tolerability compared to polytherapy, with studies showing no significant differences in quality of life outcomes between the two approaches [10,11]. However, polytherapy may be considered for drug-resistant cases, though it carries a higher risk of adverse effects such as fatigue, somnolence, and cognitive impairment [12,13]. The data indicate a varying frequency of polytherapy use in pediatric individuals with epilepsy, but it may be as high as 40–50% [12]. Guidelines recommend optimizing monotherapy before adding a second antiepileptic drug, reserving polytherapy for refractory seizures while monitoring for drug interactions and toxicity [10].

Long-term therapy with ASMs increases oxidative stress, which may be one of the mechanisms contributing to teratogenicity [14,15]. Previous data have shown that ASMs in therapeutic doses can adversely alter the redox balance in humans [16,17]. One of the strongest antioxidants in the human body is glutathione, which plays a key role in reducing oxidative stress, regulating immunity, and neutralizing free radicals (FR) generated in metabolic processes or other factors that can damage cells. Among the main fat-soluble antioxidants, retinol and tocopherols may show a significant impact on epilepsy development. Tocopherols protect the integrity of the cell membrane from FR and prevent lipid peroxidation in cell membranes [18]. The well-documented antioxidant properties of retinol are based on the effective reduction of lipid peroxidation, thereby reducing the risk of heart disease [19]. Previous data demonstrated some differences in retinol concentrations in epileptic patients depending on their age; patients over 6 years of age had higher retinol levels than younger children [20].

The present study aimed to provide an initial comparison of the concentration of selected antioxidants, including glutathione, tocopherols (α- and γ-), and retinol, between children with epilepsy treated with polytherapy for at least 6 months and control children without epilepsy. In the study, we also compared antioxidant levels between sex and age subgroups of individuals with epilepsy.

## 2. Materials and Methods

### 2.1. Study Groups

In the study, 44 individuals were recruited from the Department of Pediatric Neurology of the Medical University of Silesia in Katowice (Poland). The study group included 21 individuals with epilepsy who were treated with polytherapy. Inclusion criteria for individuals with epilepsy were as follows: diagnosis of epilepsy based on the clinical picture and the results of additional tests, especially electroencephalography (EEG) and neuroimaging tests such as magnetic resonance imaging (MRI) and/or computed tomography (CT) of the head; patient’s age below 18 years; polytherapy with ASMs for at least 6 months. Exclusion criteria from the study group contained non-epileptic seizures, no reliable diagnosis of epilepsy, diagnosis of cardiovascular diseases (arterial ischemic stroke, hypertension, heart diseases). There was no information about active epileptic seizures at the time of blood collection. There were also no individuals with epilepsy with a special diet in the study.

The control group (*n* = 23) was recruited among individuals without seizures, hospitalized with mild to moderate head injuries. Criteria for exclusion from the control group included history of seizure events (epileptic and non-epileptic), as well as treatment with ASMs for reasons other than epilepsy (i.e., behavioral problems, sleep disorders, and migraine.

Written informed consent was obtained from each patient’s parents. The research was approved by the Ethics Committee of the Medical University of Silesia in Katowice (approval number PCN/0022/KB1/43/IV/20/21 issued on 7 December 2021).

### 2.2. Analyses in Subgroups of Epileptic Patients

Analyzed parameters were assessed in the sex subgroups (i.e., comparison was performed between female individuals with epilepsy vs. male individuals with epilepsy) and in the age subgroups (i.e., comparison was made between individuals with epilepsy below 6 years of age vs. individuals with epilepsy above 6 years of age).

Even though the total number of individuals with epilepsy was low (*n* = 21) and therefore the vast majority of drug combinations occurred in a single individual, we were able to create subgroups according to the combination of ASMs used. We distinguished two subgroups: the first subgroup treated with the combination of VPA and LEV (*n* = 6) and the second subgroup, in which individuals took various other combinations of 2, 3, or 4 drugs (*n* = 15)—ASM combinations other than VPA and LEV (i.e., a comparison was made between VPA and LEV vs. combinations other than VPA and LEV).

### 2.3. Determination of Antioxidants

The concentrations of selected antioxidants were determined in serum samples using high-performance liquid chromatography (HPLC). The concentration of glutathione was determined by using a method described previously [21]. Tri-n-butylphosphine and 7-fluorobenzene-2-oxa-1,3-diazole-4-sulfonate (SBD-F) were added to 200 µL of blood serum. The sample was separated on a LiChrospher 100 RP 18 chromatographic column, 250 mm × 4 mm ID, 5 µm (Merck, Darmstadt, Germany). Chromatographic separation was carried out in the reverse phase, in a concentration gradient. Phase A was an acetate buffer with pH = 4.0, and phase B was a phosphate-buffer solution with pH = 6.0. Detection was performed using a fluorescence detector (excitation wavelength 385 nm; emission wavelength 515 nm). The calibration curve for glutathione was determined in the concentration range of 0.5–15 µmol/L.

In turn, serum levels of retinol and tocopherols were assessed according to the method described by Sobczak et al. [22]. Ethanol, ethyl acetate-butanol mixture, and solid sodium sulfate were added to 100 µL of the sample according to the scheme described in the publication. The samples obtained in this way were subsequently determined by HPLC with UV detection. Retinol analysis was performed at an excitation wavelength of 300 nm and emission wavelength of 480 nm, and tocopherols at an excitation wavelength of 285 nm and emission wavelength of 325 nm. Calibration curves for retinol, α-tocopherol, and γ-tocopherol were determined. Standard solutions of the vitamins to be determined were prepared in the following concentration range: 0.05–300 µmol/L in the case of retinol; 0.01–11 µmol/L in the case of α-tocopherol; and 0.1–38 µmol/L in the case of γ-tocopherol.

### 2.4. Levels of Lipid Parameters

For the present analyses, we also used serum levels of lipid parameters (i.e., total cholesterol (TC) and triglycerides (TG)), which were determined spectrophotometrically using commercial kits [23], to establish ratios for tocopherol/TC, tocopherol/TG, and tocopherol/TC + TG. These ratios were used to normalize serum concentration of vitamin E and correct vitamin-free lipoproteins [24].

### 2.5. Statistical Analyses

The STATISTICA 13 software (STATSOFT; Statistica, Tulsa, OK, USA) was used for statistical evaluation of the data. The mean (M) values and standard deviations (SDs) were estimated for continuous variables, while for the categorical variables, the absolute numbers (*n*) and relative numbers (%) were estimated. The normality of the data was verified with the Shapiro–Wilk test and based on the visual assessment of the histograms. Due to the small size of the groups, comparisons of the quantitative data between analyzed patients and controls were performed using a non-parametric *U* Mann–Whitney test throughout the study. A stochastic independence test with Yates’s correction was used to compare the categorical variables between study groups and/or subgroups. In addition, correlation coefficients between analyzed parameters were estimated. The result was considered statistically significant when the p-value was below 0.05.

## 3. Results

### 3.1. Characteristics of the Analyzed Groups

The average age of individuals with epilepsy was 7.1 ± 4.4 years and 7.4 ± 3.9 years in the control group. Females constituted 38% in the group with epilepsy (*n* = 8), and 30% in the control group (*n* = 7). Individuals with epilepsy were treated with ASMs for 4.6 ± 4.3 years on average.

The most frequent morphology of seizures in the group with epilepsy was generalized seizures in 14 individuals (67%), polymorphic seizures in 5 individuals (24%), myoclonic seizures in 5 individuals (24%), and consciousness disturbances in 4 individuals (19%). Further, 15 out of 21 individuals with epilepsy had an unknown etiology of epilepsy; in 4 out of 21, epilepsy was structural; and 2 individuals presented with genetic epilepsy due to Prader–Willi syndrome and Dravet syndrome.

Among individuals with epilepsy, 13 out of 21 (62%) were taking 2 ASMs, 7 out of 21 (33%) were taking 3 ASMs, and only 1 child was taking a combination of 4 ASMs (5%). Among individuals with combinations of two ASMs, the most common combination included VPA and LEV, which was used in 6 out of 13 children (46%). Table 1 demonstrates the general and biochemical characteristics of the individuals with epilepsy. No differences in terms of age, number of ASMs taken, TC, TG, α-tocopherol, or glutathione levels between male and female individuals with epilepsy were demonstrated. However, there were tendencies to higher levels of retinol in female individuals with epilepsy and to a higher level of γ-tocopherol in male individuals with epilepsy, but the results were on the bound of significance (*p* = 0.076 and *p* = 0.053, respectively).

No differences were found in the concentrations of the analyzed parameters between distinguished age subgroups.

### 3.2. Comparison of Concentrations of Selected Antioxidants Between the Study Group and the Control Group

The differences in antioxidants between the individuals with epilepsy and the control group were assessed. The significant difference was demonstrated only in the glutathione concentration between individuals with epilepsy and the control group. Individuals without epilepsy had higher glutathione than individuals with epilepsy (2.4 ± 1.2 µmol/L vs. 1.5 ± 0.3 µmol/L, respectively, *p* < 0.001). No differences were observed in the levels of retinol and α- and γ-tocopherol between the groups. However, the ratios of both α-tocopherol/glutathione and γ-tocopherol/glutathione were found to be significantly higher in the individuals with epilepsy than in the control group. Detailed results in the analyzed antioxidant levels between the study and control groups, depending on sex, are shown in Table 2.

### 3.3. Analysis of Tocopherol/Lipid Ratios Between the Study and Control Groups

Table 3 presents the ratios of tocopherol/TC, tocopherol/TG, and tocopherol/TC + TG between the controls and individuals with epilepsy. No statistically significant differences were found between the analyzed subgroups.

### 3.4. Analysis of Concentrations of Selected Antioxidants Depending on the ASMs Taken

Individuals with epilepsy were treated with various ASMs, and 11 patients took a combination of drugs that was not repeated in other patients. Thus, we were able to divide patients into two subgroups according to the ASMs combination taken, i.e., one subgroup included patients treated with the VPA and LEV combination (*n* = 6), and the other patients with other ASMs combinations (*n* = 15). VPA and LEV patients had significantly higher levels of both retinol and glutathione (*p* = 0.047 and *p* = 0.003, respectively) than the second ASMs subgroup. When compared to the control group, children treated with a combination of ASMs other than VPA and LEV also had significantly lower levels of glutathione (*p* < 0.001). Such a difference was not observed for retinol level. Table 4 demonstrates mean concentrations of analyzed antioxidants in groups of patients depending on ASM combinations.

### 3.5. Correlations Between Age and TC and the Analyzed Antioxidants

Intercorrelations between the analyzed antioxidants, i.e., glutathione, retinol, α- and γ-tocopherol, and age, and total cholesterol were analyzed. In the group of individuals with epilepsy, we found a positive mutual correlation between γ-tocopherol and glutathione (r = 0.451, *p* = 0.040). In turn, the positive correlation between retinol and γ-tocopherol was observed in the control group (r = 0.531, *p* = 0.009).

In turn, in the case of the correlation of antioxidants with age in the individuals with epilepsy, only a negative relationship was observed between α-tocopherol and age (r = −0.505, *p* = 0.019). The younger the age, the higher the level of α-tocopherol. However, no correlation was found between antioxidants and TC in the group with epilepsy. In the control group, the levels of retinol and γ-tocopherol were positively correlated with age (r = 0.573, *p* = 0.004 and r = 0.461, *p* = 0.027, respectively), while only the level of retinol was positively correlated with the TC level (r = 0.491, *p* = 0.017). The exact data on correlation coefficients between selected variables are shown in Table 5.

## 4. Discussion

In the present study, significantly lower levels of glutathione were observed in the individuals with epilepsy compared to the control group. Simultaneously, no differences in glutathione levels between sex and age subgroups among individuals with epilepsy were found. However, after dividing individuals with epilepsy based on the ASMs used, those with epilepsy taking ASM combinations other than VPA and LEV had a lower level of both retinol and glutathione than individuals taking VPA and LEV treatment. In addition, individuals with epilepsy on an ASM combination other than VPA and LEV also had a lower level of glutathione compared to the controls

Oxidative stress is involved in the pathogenesis of many central nervous system disorders (CNS), including epilepsy. Numerous data indicate increased oxidative stress in individuals with epilepsy taking ASMs [18,25,26,27,28]. However, there are also data that suggest that oxidative stress may be caused by epileptic seizures themselves, not ASMs [29]. Since glutathione protects cells from oxidative damage as a free radical scavenger, its deficiency may increase oxidative stress by initiating cell death in various neurons [30]. Some ASMs can reduce plasma glutathione levels, reflecting treatment-related oxidative stress [31]. However, data on the antioxidants’ (i.e., glutathione, retinol, tocopherol) levels in children or young individuals with epilepsy on ASMs therapy are scarce and often give contradictory results [29,32,33,34]. It was observed that the level of glutathione was almost two-fold lower in 35 young individuals with refractory epilepsy from Turkey compared to controls [32]. In turn, Menon et al. [29] showed that ASM therapy did not significantly affect the levels of glutathione in young individuals (mean age of 26.23 ± 10.6 years, the mean duration of illness was 7 ± 6 years) compared to untreated individuals. On the contrary, a study by Ramazan et al. [33] reported higher glutathione levels in VPA and lamotrigine individuals with epilepsy than in controls.

In our study, no significant differences in retinol levels were found between the group with epilepsy and the controls. Similarly to our results, in the study by Bakhtiari et al. [34], the concentration of retinol did not differ between individuals with febrile seizures and the control group. On the other hand, Menon et al. [29] observed significantly lower tocopherol levels in young individuals with epilepsy compared to controls, but the difference especially concerned untreated ASMs individuals vs. controls.

Our study demonstrates that in individuals with epilepsy, the level of α-tocopherol decreases with age. In turn, in the control group, the levels of retinol and γ-tocopherol were positively correlated with age. As for the dependence of glutathione levels on age, we did not observe such changes in our research groups. Previously, much data have shown that the concentration of antioxidants, i.e., retinol and tocopherols, changes with age [20,35,36]. In a group of 1000 healthy individuals aged between 10.0 and 71.3 years, Al-Saleh et al. [36] demonstrated that people between 10 and 20 years had the lowest serum levels of both dl-α-tocopherol and all-trans-retinol compared to older subjects. An increase in retinol levels with age was also demonstrated in a large group of 342 healthy Canadian children (aged 1 day to 19 years of age) [37]. Nau et al. [20] observed lower mean plasma retinol levels in pediatric individuals with epilepsy from 0 to 6 years of age than in children above 6 years of age. In addition, the authors found significantly higher mean plasma retinol in younger-aged children with epilepsy than in the similarly aged controls. On the contrary, data from young adults from Iraq (mean age of 20.94 ± 10.63 years) showed no correlation of tocopherol with age [38].

Studies on diet in epilepsy do not indicate that glutathione or retinol and tocopherols obtained from the diet have a positive or negative effect on the severity of epileptic seizures. Data from both animal models and randomized trials show some promising but often contradictory results. Ambrogini et al. [39] suggested that α-tocopherol disrupts epileptogenesis in a rat model by targeting neuroinflammation, oxidative stress, and maladaptive plasticity, while preserving blood–brain barrier integrity. The study highlighted α-tocopherols potential as an adjunct therapy to prevent chronic epilepsy development post-status epilepticus. On the other hand, retinol showed no effect on clonic and tonic seizures induced by pentylenetetrazole in mice [40].

In the study by Ogunmekan et al. [41], the addition of 400 IU/day of D-alpha-tocopheryl acetate to ASMs used by children with epilepsy resulted in a significant reduction in seizures in 10 out of 12 patients. Tocopherols were demonstrated to reduce the frequency of seizures, and their use in combination with ascorbic acid has been considered as an adjunctive therapy in individuals with drug-resistant epilepsy [37].

In our control group, the level of retinol was the only antioxidant positively correlated with the TC level. We also established ratios of tocopherol/TC and/or TG; however, these did not differ between groups with epilepsy and controls. In turn, α and γ-tocopherol/glutathione ratios appeared to be higher in individuals with epilepsy compared to the control group. Since tocopherol is a fat-soluble vitamin bound to lipoproteins that circulates in the bloodstream, its serum concentration is strongly influenced by lipid levels. Measuring only the absolute concentration of tocopherol can be misleading, especially in individuals with abnormal lipid profiles, such as pediatric patients. Adjusting tocopherol for lipid concentrations using the ratios of tocopherol/TC and tocopherol/(TC + TG) may provide a more accurate assessment of tocopherol status and its adequacy in the body. Previously, lower tocopherol/(TC + TG) ratios were significantly associated with the presence and severity of metabolic syndrome and its components, as well as markers of inflammation and diabetes in individuals from Iran [24].

Our study has certain limitations. The number of pediatric individuals with epilepsy was rather low (*n* = 21). This may result from different issues. First, children are a very difficult study group, mainly due to the parents’ concerns and thus the difficulties in obtaining parental consent for the study. Second, our patients were recruited during the first and the second waves of the COVID-19 pandemic, which resulted in some problems in the functioning of the hospital, as well as the parents’/guardians’ attitude. However, the number of individuals with epilepsy we gathered did not significantly differ from the number of polytherapy patients in other published papers [26,28]. In this study, we also did not have specific information regarding the use of antioxidants in the diet of children treated for epilepsy.

However, since there is little research in this area, our data may constitute an incentive for further, broader research on the relationship between the level of antioxidants in children with epilepsy and the possibility of using an appropriate diet. Recent randomized clinical trials in patients with epilepsy show that daily multivitamin treatment for 6 months led to a significant reduction in the frequency of seizures [42].

## 5. Conclusions

Our results indicate that long-term ASM therapy may reduce serum glutathione levels, which may indicate oxidative stress developing during therapy. In contrast, the levels of retinol and tocopherol did not differ between individuals with and without epilepsy. Particularly low levels of both glutathione also retinol were observed in children with epilepsy treated with drug combinations other than VPA and LEV. The problem of exposure to oxidative stress is particularly important in the pediatric population due to the developing organism of a child with epilepsy and the distant effects of oxidative stress, which can accumulate and occur in adulthood. Therefore, the results of our study can certainly contribute to more precise observation of children with epilepsy for antioxidant deficiency, because in some cases, supplementation of some of them can be considered.

## Figures and Tables

**Table 1:** General and biochemical characteristics of the individuals with epilepsy depending on sex and ASMs combinations.

Variable	Total Group with Epilepsy *N* = 21	Sex Subgroups		*p* *	Age Subgroups		*p* **
		Male *N* = 13 (62%)	Female *N* = 8 (38%)		<6 Years of Age *N* = 11 (52%)	>6 Years of Age *N* = 10 (48%)	
Age (years), M ± SD	7.1 ± 4.4	7.3 ± 3.7	6.8 ± 5.5	0.362	3.7 ± 0.7	10.9 ± 3.4	**<0.001**
Number of ASMs taken, n (%)				0.999			0.556
Two	13 (62)	8 (61)	5 (62)		7 (64)	6 (60)	
Three	7 (33)	5 (38)	2 (25)		4 (36)	3 (30)	
Four	1 (5)	0 (0)	1 (12)		0 (0)	1 (10)	
TC (mg/dL), M ± SD	129 ± 27	126 ± 27	132 ± 27	0.500	129 ± 24	128 ± 31	0.647
TG (mg/dL), M ± SD	104 ± 47	112 ± 49	90 ± 44	0.860	93 ± 46	116 ± 48	0.274
Retinol (µmol/L), M ± SD	0.47 ± 0.13	0.44 ± 0.11	0.52 ± 0.15	0.076	0.44 ± 0.15	0.51 ± 0.09	0.275
α-tocopherol (µmol/L), M ± SD	9.2 ± 3.3	9.6 ± 2.8	8.5 ± 4.2	0.414	10.3 ± 3.2	8.0 ± 3.2	0.084
γ-tocopherol (µmol/L), M ± SD	0.51 ± 0.34	0.61 ± 0.36	0.35 ± 0.23	0.053	0.48 ± 0.37	0.55 ± 0.31	0.418
Glutathione (µmol/L), M ± SD	1.5 ± 0.3	1.5 ± 0.4	1.4 ± 0.1	0.500	1.5 ± 0.4	1.5 ± 0.3	0.698

*—comparison between male and female subgroups; **—comparison between age subgroups; M—mean; SD—standard deviation; ASMs—anti-seizure medications; TC—total cholesterol; TG—triglycerides. Statistical differences are in bold.

**Table 2:** Differences in concentrations of selected antioxidants as well as their ratios between individuals with epilepsy and control children.

Variable	Total *N* = 44	Individuals with Epilepsy *N* = 21	Control Group *N* = 23	*p*
Retinol (µmol/L), M ± SD	0.46 ± 0.13	0.47 ± 0.13	0.44 ± 0.13	0.388
α-tocopherol (µmol/L), M ± SD	9.3 ± 2.8	9.2 ± 3.4	9.4 ± 2.3	0.780
γ-tocopherol (µmol/L), M ± SD	0.44 ± 0.27	0.51 ± 0.34	0.38 ± 0.17	0.388
Glutathione (µmol/L), M ± SD	2.0 ± 1.0	1.5 ± 0.3	2.4 ± 1.2	**<0.001**
α-tocopherol/γ-tocopherol ratio, M ± SD	31 ± 36	25 ± 17	36 ± 47	0.252
α-tocopherol/glutathione ratio, M ± SD	5.6 ± 2.5	6.5 ± 2.8	4.7 ± 1.9	**0.042**
γ-tocopherol/glutathione ratio, M ± SD	0.26 ± 0.19	0.35 ± 0.23	0.18 ± 0.10	**0.004**

M—mean; SD—standard deviation. Significant differences are in bold.

**Table 3:** Differences in ratios of tocopherol/lipids between individuals with epilepsy and controls.

Variable	Total *N* = 44	Individuals with Epilepsy *N* = 21	Control Group *N* = 23	*p*
α-tocopherol/TC ratio, M ± SD	0.074 ± 0.024	0.072 ± 0.024	0.075 ± 0.025	0.925
α-tocopherol/TG ratio, M ± SD	0.121 ± 0.070	0.109 ± 0.065	0.133 ± 0.070	0.301
α-tocopherol/TC + TG ratio, M ± SD	0.044 ± 0.018	0.041 ± 0.016	0.046 ± 0.019	0.347
γ-tocopherol/TC ratio, M ± SD	0.003 ± 0.002	0.004 ± 0.003	0.003 ± 0.001	0.152
γ-tocopherol/TG ratio, M ± SD	0.005 ± 0.004	0.005 ± 0.003	0.005 ± 0.004	0.796
γ-tocopherol/TC + TG ratio, M ± SD	0.002 ± 0.001	0.002 ± 0.001	0.002 ± 0.001	0.411

M—mean; SD—standard deviation; TC—total cholesterol; TG—triglycerides.

**Table 4:** Concentrations of analyzed antioxidants in individuals with epilepsy depending on ASMs combinations.

Variable	Total Group with Epilepsy *N* = 21	A Combination of VPA and LEV of ASMs Used *N* = 6 (29%)	A Combination of ASMs Other Than VPA and LEV *N* = 15 (71%)	*p*
Retinol (µmol/L), M ± SD	0.47 ± 0.13	0.56 ± 0.07	0.44 ± 0.13	**0.047**
α-tocopherol (µmol/L), M ± SD	9 ± 3	8 ± 2	10 ± 4	0.331
γ-tocopherol (µmol/L), M ± SD	0.51 ± 0.34	0.56 ± 0.23	0.49 ± 0.37	0.259
Glutathione (µmol/L), M ± SD	1.5 ± 0.3	1.8 ± 0.3	1.3 ± 0.3	**0.003**

M—mean; SD—standard deviation; ASMs—anti-seizure medications; VPA—valproic acid; LEV—levetiracetam. Statistical differences are in bold.

**Table 5:** Correlation coefficients between age and the levels of total cholesterol and antioxidants in the analyzed groups.

	Individuals with Epilepsy				Control Group			
	Age (Years)		TC (mg/dL)		Age (Years)		TC (mg/dL)	
Antioxidants	r	*p*	r	*p*	r	*p*	r	*p*
Retinol (µmol/L), M ± SD	0.128	0.580	0.065	0.780	0.573	0.004	0.491	0.017
α-tocopherol (µmol/L), M ± SD	−0.505	0.019	0.274	0.229	−0.025	0.910	0.132	0.548
γ-tocopherol (µmol/L), M ± SD	0.169	0.463	−0.087	0.708	0.461	0.027	0.276	0.203
Glutathione (µmol/L), M ± SD	−0.076	0.742	−0.087	0.708	0.031	0.892	−0.032	0.887

M—mean; SD—standard deviation; TC—total cholesterol; r—correlation coefficient.

## Data Availability

The data presented in this study are available on request in the Department of Basic Biomedical Science, Faculty of Pharmaceutical Sciences, Medical University of Silesia in Katowice (Poland). The data are not publicly available due to privacy restrictions.

## References

[1] Guekht A., Brodie M., Secco M., Li S., Volkers N., Wiebe S. (2021). The Road to a World Health Organization Global Action Plan on Epilepsy and Other Neurological Disorders. Epilepsia.

[2] Epilepsy: A Public Health Imperative World Health Organization 2019. https://www.who.int/publications/i/item/epilepsy-a-public-health-imperative.

[3] Cardenas-Rodriguez N., Huerta-Gertrudis B., Rivera-Espinosa L., Montesinos-Correa H., Bandala C., Carmona-Aparicio L., Coballase-Urrutia E. (2013). Role of Oxidative Stress in Refractory Epilepsy: Evidence in Patients and Experimental Models. Int. J. Mol. Sci..

[4] Vu L.C., Piccenna L., Kwan P., O’Brien T.J. (2018). New-Onset Epilepsy in the Elderly. Br. J. Clin. Pharmacol..

[5] Baulac M., De Boer H., Elger C., Glynn M., Kälviäinen R., Little A., Mifsud J., Perucca E., Pitkänen A., Ryvlin P. (2015). Epilepsy Priorities in Europe: A Report of the ILAE-IBE Epilepsy Advocacy Europe Task Force. Epilepsia.

[6] Puttachary S., Sharma S., Stark S., Thippeswamy T. (2015). Seizure-induced oxidative stress in temporal lobe epilepsy. BioMed Res. Int..

[7] Sarecka-Hujar B., Szołtysek-Bołdys I., Kopyta I., Dolińska B., Sobczak A. (2019). Concentrations of the Selected Biomarkers of Endothelial Dysfunction in Response to Antiepileptic Drugs: A Literature Review. Clin. Appl. Thromb. Hemost..

[8] Anwar H., Khan Q.U., Nadeem N., Pervaiz I., Ali M., Cheema F.F. (2020). Epileptic seizures. Discoveries.

[9] Mlinar S., Petek D., Cotič Ž., Mencin Čeplak M., Zaletel M. (2016). Persons with Epilepsy: Between Social Inclusion and Marginalisation. Behav. Neurol..

[10] St Louis E.K., Rosenfeld W.E., Bramley T. (2009). Antiepileptic drug monotherapy: The initial approach in epilepsy management. Curr. Neuropharmacol..

[11] Henry Daniel Raj T., Sylvia A., Chidambaranathan S., Nirmala P. (2017). Monotherapy and polytherapy in Paediatric seizures: A prospective, observational study in a tertiary care teaching hospital. IAIM.

[12] Kopciuch D., Kus K., Fliciński J., Steinborn B., Winczewska-Wiktor A., Paczkowska A., Zaprutko T., Ratajczak P., Nowakowska E. (2022). Pharmacovigilance in Pediatric Patients with Epilepsy Using Antiepileptic Drugs. Int. J. Environ. Res. Public Health.

[13] Egunsola O., Sammons H.M., Whitehouse W.P. (2016). Monotherapy or polytherapy for childhood epilepsies?. Arch. Dis. Child..

[14] Liu L., Wells P.G. (1995). DNA oxidation as a potential molecular mechanism mediating drug-induced birth defects: Phenytoin and structurally related teratogens initiate the formation of 8-hydroxy-2′-deoxyguanosine in vitro and in vivo in murine maternal hepatic and embryonic tissues. Free Radic. Biol. Med..

[15] Martinc B., Grabnar I., Milosheska D., Lorber B., Vovk T. (2024). A Cross-Sectional Study Comparing Oxidative Stress in Patients with Epilepsy Treated with Old and New Generation Antiseizure Medications. Medicina.

[16] Aycicek A., Iscan A. (2007). The effects of carbamazepine, valproic acid and phenobarbital on the oxidative and antioxidative balance in epileptic children. Eur. Neurol..

[17] Michoulas A., Tong V., Teng X.W., Chang T.K.H., Abbott F.S., Farrell K. (2006). Oxidative stress in children receiving valproic acid. J. Pediatr..

[18] Sudha K., Rao A.V., Rao A. (2001). Oxidative stress and antioxidants in epilepsy. Clin. Chim. Acta.

[19] Palace V.P., Khaper N., Qin Q., Singal P.K. (1999). Antioxidant potentials of vitamin A and carotenoids and their relevance to heart disease. Free Radic. Biol. Med..

[20] Nau H., Tzimas G., Mondry M., Plum C., Spohr H.-L. (1995). Antiepileptic drugs alter endogenous retinoid concentrations: A possible mechanism of teratogenesis of anticonvulsant therapy. Life Sci..

[21] Teerlink T. (2005). Determination of the Endogenous Nitric Oxide Synthase Inhibitor Asymmetric Dimethylarginine in Biological Samples by HPLC. Hypertens. Methods Protoc..

[22] Sobczak A., Skop B., Kula B. (1999). Simultaneous Determination of Serum Retinol and α-and γ-Tocopherol Levels in Type II Diabetic Patients Using High-Performance Liquid Chromatography with Fluorescence Detection. J. Chromatogr. B Biomed. Sci. Appl..

[23] Sarecka-Hujar B., Szołtysek-Bołdys I., Kopyta I. (2022). Serum Levels of Lipids and Selected Aminothiols in Epileptic Children—A Pilot Case-Control Study. Brain Sci..

[24] Barzegar-Amini M., Khorramruz F., Ghazizadeh H., Sahebi R., Mohammadi-Bajgyran M., Mohaddes Ardabili H., Tayefi M., Darroudi S., Moohebati M., Heidari-Bakavoli A. (2021). Association between serum Vitamin E concentrations and the presence of Metabolic Syndrome: A population-based cohort study. Acta Biomed..

[25] Hamed S.A., Abdellah M.M., El-Melegy N. (2004). Blood Levels of Trace Elements, Electrolytes, and Oxidative Stress/Antioxidant Systems in Epileptic Patients. J. Pharmacol. Sci..

[26] Turkdogan D., Toplan S., Karakoc Y. (2002). Lipid Peroxidation and Antioxidative Enzyme Activities in Childhood Epilepsy. J. Child Neurol..

[27] Huang S., Chen Y., Wang Y., Pan S., Lu Y., Gao W., Hu X., Fang Q. (2024). Diet-Derived Circulating Antioxidants and Risk of Epilepsy: A Mendelian Randomization Study. Front. Neurol..

[28] Elkhayat H.A., Hamed H.M., Shouman M.G., Elagouza I.A., Mekkawy L.H. (2018). Influence of Conventional Antiepileptic Drugs on Glutathione-S-Transferase and Lipid Peroxidation in Children with Idiopathic Epilepsy. Bull. Natl. Res. Cent..

[29] Menon B., Ramalingam K., Kumar R.V. (2014). Low Plasma Antioxidant Status in Patients with Epilepsy and the Role of Antiepileptic Drugs on Oxidative Stress. Ann. Indian Acad. Neurol..

[30] Cárdenas-Rodriguez N., Coballase-Urrutia E., Pérez-Cruz C., Montesinos-Correa H., Rivera-Espinosa L., Sampieri A., Carmona-Aparicio L. (2014). Relevance of the Glutathione System in Temporal Lobe Epilepsy: Evidence in Human and Experimental Models. Oxid. Med. Cell. Longev..

[31] Ono H., Sakamoto A., Sakura N. (2000). Plasma Total Glutathione Concentrations in Epileptic Patients Taking Anticonvulsants. Clin. Chim. Acta.

[32] Yilgor A., Demir C. (2024). Determination of oxidative stress level and some antioxidant activities in refractory epilepsy patients. Sci. Rep..

[33] Çoban Ramazan D., Anadol Ü., Yalçın A.D., Yalçın A.S. (2019). Plasma homocysteine and aminothiol levels in idiopathic epilepsy patients receiving antiepileptic drugs. Turk. J. Biochem..

[34] Bakhtiari E., Heydarian F., Azmoudeh F., Kaffashbashi M., Heidarian M. (2023). Serum Level of Vitamin A in Febrile Children with and without Seizure: A Comparative Study. Heliyon.

[35] Stuetz W., Weber D., Dollé M.E.T., Jansen E., Grubeck-Loebenstein B., Fiegl S., Toussaint O., Bernhardt J., Gonos E.S., Franceschi C. (2016). Plasma Carotenoids, Tocopherols, and Retinol in the Age-Stratified (35–74 Years) General Population: A Cross-Sectional Study in Six European Countries. Nutrients.

[36] Al-Saleh I., El-Doush I., Billedo G. (2007). Age and gender-related reference values for serum dl-alpha-tocopherol and all-trans-retinol levels in Saudi population. Int. J. Vitam. Nutr. Res..

[37] Raizman J.E., Cohen A.H., Teodoro-Morrison T., Wan B., Khun-Chen M., Wilkenson C., Bevilaqua V., Adeli K. (2014). Pediatric Reference Value Distributions for Vitamins A and E in the CALIPER Cohort and Establishment of Age-Stratified Reference Intervals. Clin. Biochem..

[38] Muslim A.T., Altememi W.H., Al-Obeidy E.S., Shareef B.Q. (2023). Vitamin E level in epileptic patients attending Neuroscience hospital in Baghdad. Iraqi New Med. J..

[39] Ambrogini P., Albertini M.C., Betti M., Galati C., Lattanzi D., Savelli D., Di Palma M., Saccomanno S., Bartolini D., Torquato P. (2018). Neurobiological Correlates of Alpha-Tocopherol Antiepileptogenic Effects and MicroRNA Expression Modulation in a Rat Model of Kainate-Induced Seizures. Mol. Neurobiol..

[40] Sayyah M., Yousefi-Pour M., Narenjkar J. (2005). Anti-epileptogenic effect of beta-carotene and vitamin A in pentylenetetrazole-kindling model of epilepsy in mice. Epilepsy Res..

[41] Ogunmekan A.O., Hwang P.A. (1989). A randomized, double-blind, placebo-controlled, clinical trial of D-alpha-tocopheryl acetate (vitamin E), as add-on therapy, for epilepsy in children. Epilepsia.

[42] Chang H.H., Sung P.-S., Liao W.C., Chang A.Y.W., Hsiao Y.-H., Fu T.-F., Huang C.-Y., Huang C.-W. (2020). An Open Pilot Study of the Effect and Tolerability of Add-on Multivitamin Therapy in Patients with Intractable Focal Epilepsy. Nutrients.

